# Supplementary material for: Roles of two types of heparan sulfate clusters in Wnt distribution and signaling in Xenopus
Source: Nat Commun. 2017 Dec 7;8:1973. doi: 10.1038/s41467-017-02076-0 (PMC5719454; doi:10.1038/s41467-017-02076-0)
Supplement: Supplementary file 1 — Supplementary Information [file 41467_2017_2076_MOESM1_ESM.pdf]

Supplementary Figures

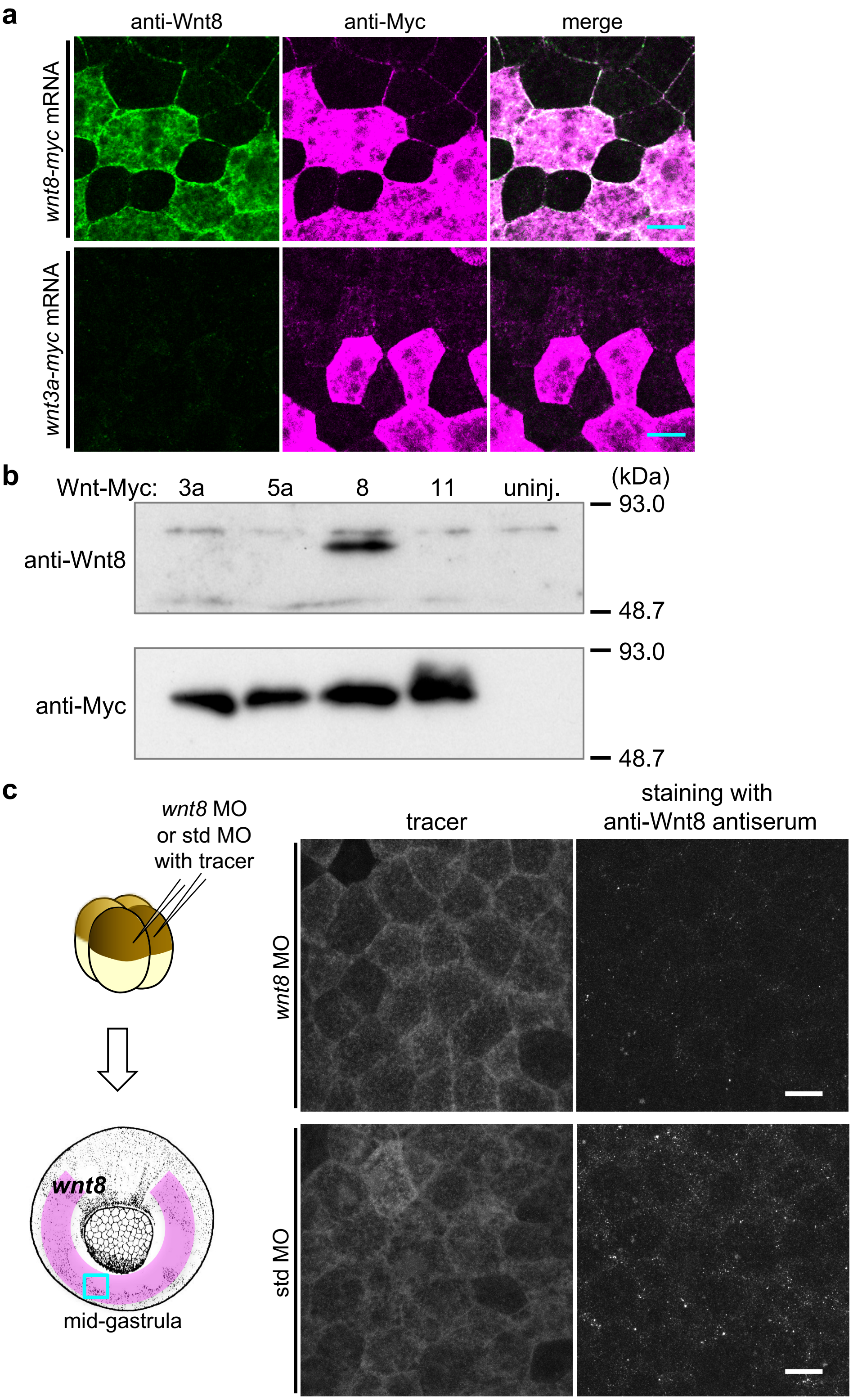

Supplementary Figure 1. Validation of anti-Wnt8 antibody.

a, Specific detection of the Wnt8 protein in *Xenopus* embryos by immunostaining with the anti-Wnt8 antiserum. Embryos were injected with mRNA for Wnt8-Myc or Wnt3a-Myc at the 4-cell stage, fixed at the gastrula stage, and subjected to immunostaining. Upper panels, staining with anti-Wnt8 antiserum mostly overlapped with that with anti-Myc antibody in Wnt8-Myc-expressing cells as well as in the intercellular space. Lower panels, no specific staining with anti-Wnt8 antiserum was detected in Wnt3a-Myc-expressing cells.

b, Specific detection of the Wnt8 protein by immunoblotting with the anti-Wnt8 antiserum. Lysates (st. 11-11.5) were prepared from *Xenopus* embryos expressing Wnt3a-, Wnt5a-, Wnt8-, or Wnt11-Myc as indicated and were subjected to immunoblotting with the anti-Wnt8 antiserum. Only Wnt8-Myc, among others, was detected as a single band, indicating the specificity of the antiserum (upper panel). The presence of Wnt-Myc proteins in lysates was assessed by blotting with the anti-Myc antibody (lower panel). The amounts of lysate per lane correspond to 1/2 and 1/4 embryo for anti-Wnt8 and anti-Myc, respectively. uninj., uninjected.

c, Reduction of endogenous Wnt8 staining with anti-Wnt8 antiserum by *wnt8* MO. *wnt8* MO or std MO (negative control) was injected with FITC-dextran (tracer) into the ventral equatorial region, which develops to the VMZ, and the superficial layer of the VMZ was observed at the mid-gastrula stage (st. 11.5). *wnt8* mRNA is expressed in the lateral and ventral mesoderm, which is underneath the superficial ectodermal layer, as indicated by the magenta region. The observed area is indicated by the cyan box in the illustration of ventral view of gastrula. *wnt8* MO but not std MO reduced immunostaining with the anti-Wnt8 antiserum, suggesting that this antiserum recognizes endogenous Wnt8.

Images are a representative of at least two independent experiments. Amounts of mRNAs (ng/embryo): 0.25 (**a**); 0.50 (**b**). Amounts of MOs, 28 ng/embryo. Scale bars, 20 μm.

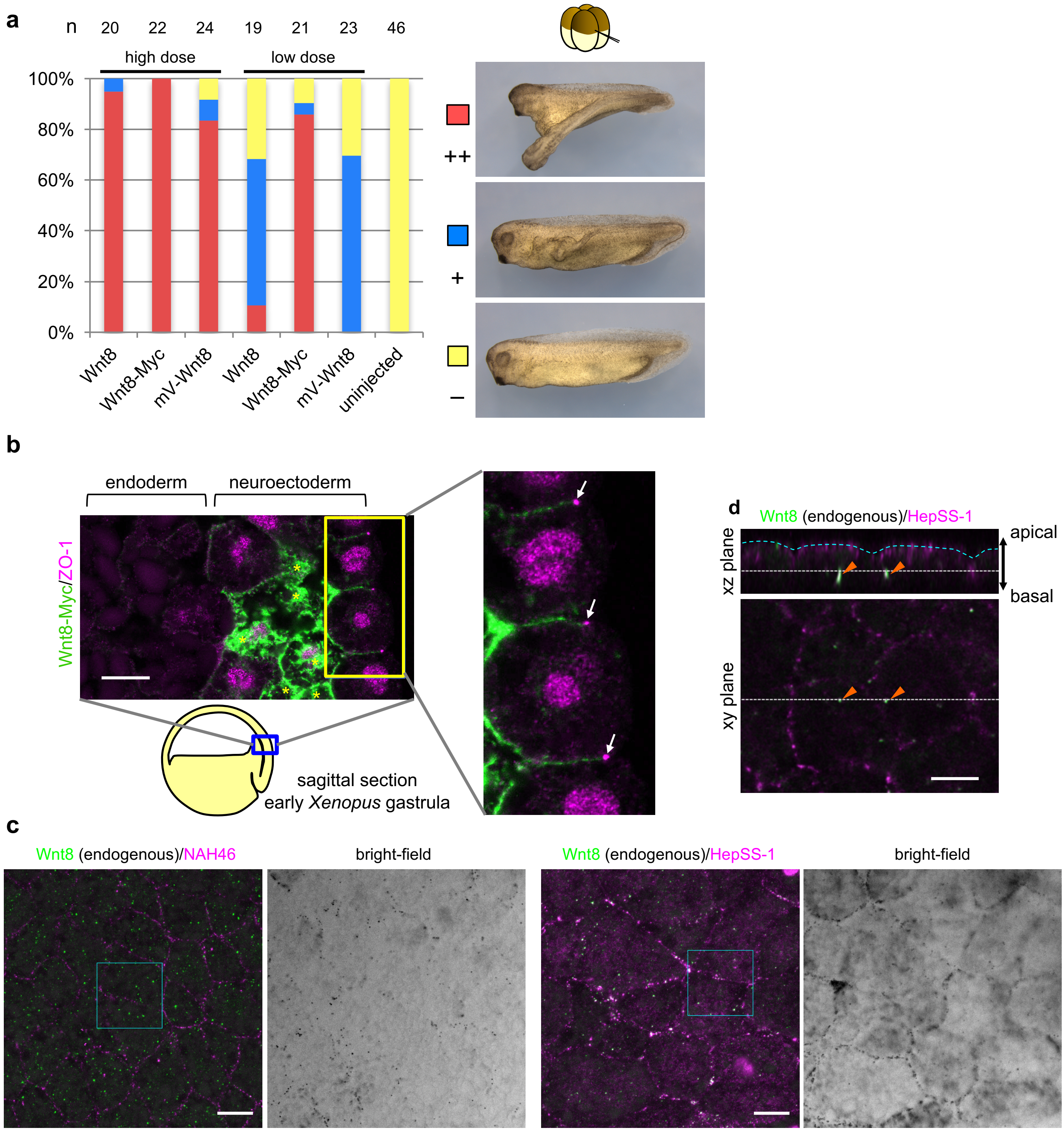

Supplementary Figure 2. Biological activities of tagged constructs and the basolateral distribution of Wnt8-Myc.

a, Biological activity of *wnt8* constructs as assayed by secondary axis formation. mRNA for each *wnt8* construct was injected into the ventral equatorial region near the midline at the 4-cell stage, and secondary axis formation was scored at the tail bud stage (st. 33/34). Phenotypes of embryos were classified into three categories as shown in the pictures: ++, secondary axis with a cement gland; +, secondary axis without a cement gland; –, no apparent secondary axis. Percentages of these categories are presented as bar graphs. Numbers of injected embryos (n) are indicated above.

b, Distribution of Wnt8-Myc in the basolateral intercellular space. Frozen sections of Wnt8-Myc expressing embryos were immunostained for Wnt8-Myc (green) and a tight junction marker ZO-1 (magenta). Source cells are as indicated (*). The section includes the neuroectoderm and the endoderm as indicated by a blue box in the schema. Right panel, a magnified image of the boxed region (yellow) in the left panel. Tight junctions are indicated by ZO-1 staining (arrows).

c, Low magnification images for the comparison of endogenous Wnt8 and the two types of HS. Boxed regions are shown in Fig. 2f,i.

d, Wnt8 puncta inside the cell. The VMZ of *Xenopus* gastrula (st. 10.5) was analysed. Wnt8 puncta, arrowheads; position of the apical cell surface, cyan dashed line; section lines, white dashed lines.

Images are a representative of at least two independent experiments. Amounts of injected mRNAs (pg/embryo): **a**, *wnt8*, 10 (high dose) or 3.3 (low dose); *mV-wnt8*, 12.5 (high dose) or 4.2 (low dose); *wnt8-myc*, 9.5 (high dose) or 3.2 (low dose). These doses are equimolar; **b**, *wnt8-myc*, 0.30. Scale bar, 20 µm.

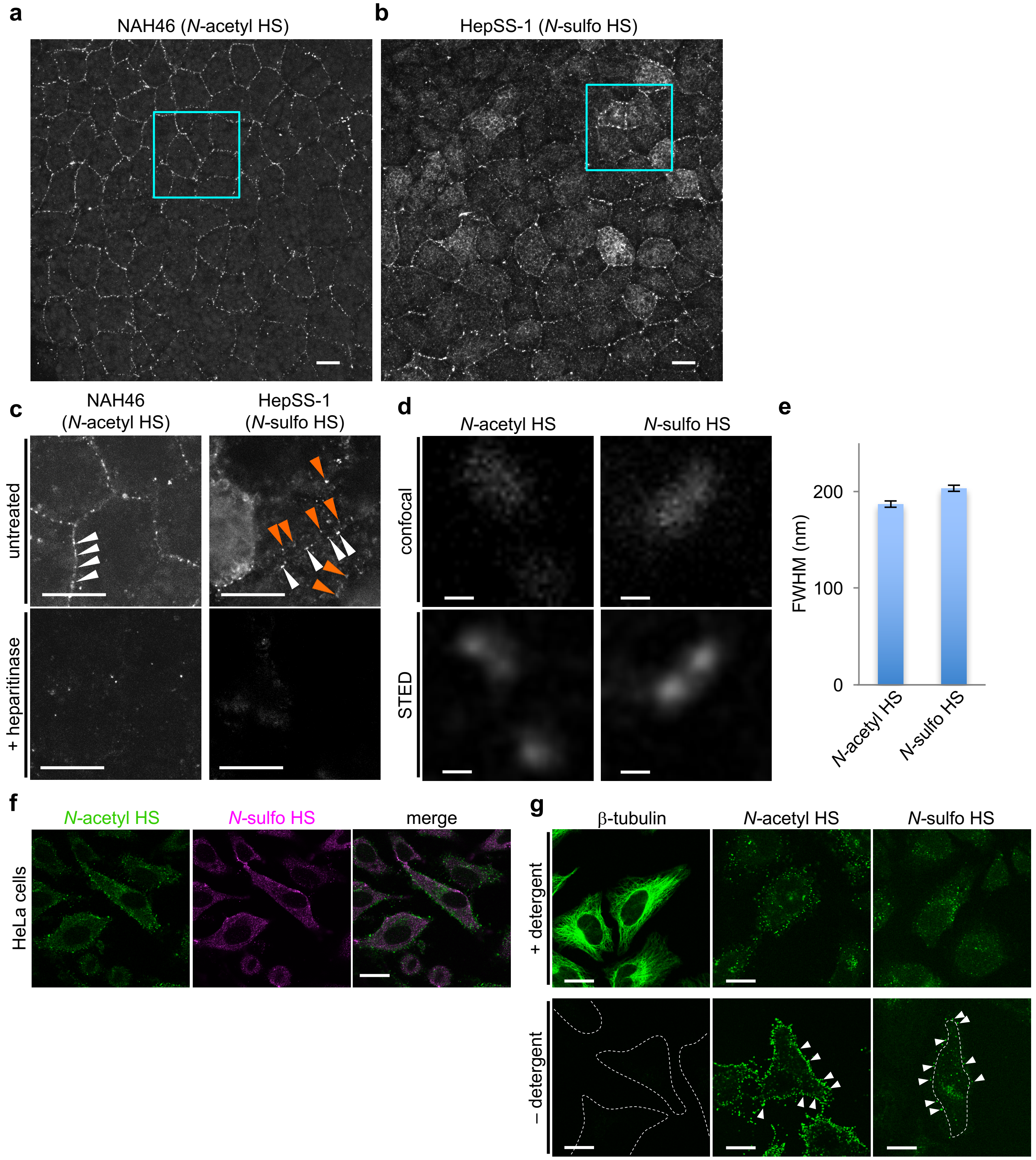

Supplementary Figure 3. Characterization of *N*-acetyl- and *N*-sulfo-rich HS clusters.

a,**b**, Immunostaining of NAH46 and HepSS-1 in a lower magnification. Staining patterns of *N*-acetyl HS are relatively uniform, whereas staining patterns of *N*-sulfo HS are relatively varied among cells. Note that similar variations have been report even within a single cell line (NIH3T3), possibly reflecting cell density and/or cell growth[1]. The blue boxes indicate the regions shown in Fig. 2e.

c, Effects of heparitinase treatment on immunostaining of HS. The animal cap region of immunostained *Xenopus* embryos was observed. Upper panels, white arrowheads, intercellular puncta; orange arrowheads, intracellular puncta. Lower panels, heparitinase was injected into the blastocoel at stage 6.5 (lower panels), confirming the specificity of immunostaining.

d, Super-resolution imaging of HS clusters using stimulated emission depletion (STED) microscopy. For comparison, confocal images are presented (upper panels). STED images showed improved spatial resolution (lower panels).

e, Quantification of full width half maxima (FWHM) of HS clusters. FWHM of each HS cluster was semi-automatically quantified along the cell boundary, using an ImageJ plug-in (see Methods). The number of analysed HS clusters: *N*-acetyl, 99; *N*-sulfo, 87. It should be noted that IgM is about 19 nm in a diameter[2], possibly causing overestimation of the sizes of two types of HS clusters.

f,**g**, *N*-acetyl- and *N*-sulfo-rich HS clusters in human HeLa cells. HeLa cells were fixed and immunostained for *N*-acetyl and *N*-sulfo HS. **f**, *N*-acetyl HS (green) and *N*-sulfo HS (magenta) showed distinct patterns. See Methods for double staining of HeLa cells. **g**, HeLa cells immunostained with or without permeabilization. Primary antibody against β-tubulin was used as a control for intracellular proteins. With permeabilization (upper panels), β-tubulin was stained inside the cells and *N*-acetyl- and *N*-sulfo-rich HS showed punctate staining. By contrast, without permeabilization (lower panels), no staining of β-tubulin was observed, but both *N*-acetyl- and *N*-sulfo-rich HS showed punctate staining (arrowheads), indicating that they are exposed to the extracellular space (cell outlines were indicated by dashed lines).

Images are a representative of at least two independent experiments. Scale bars, 20 μm (**a-c**,**f**,**g**); 200 nm (**d**).

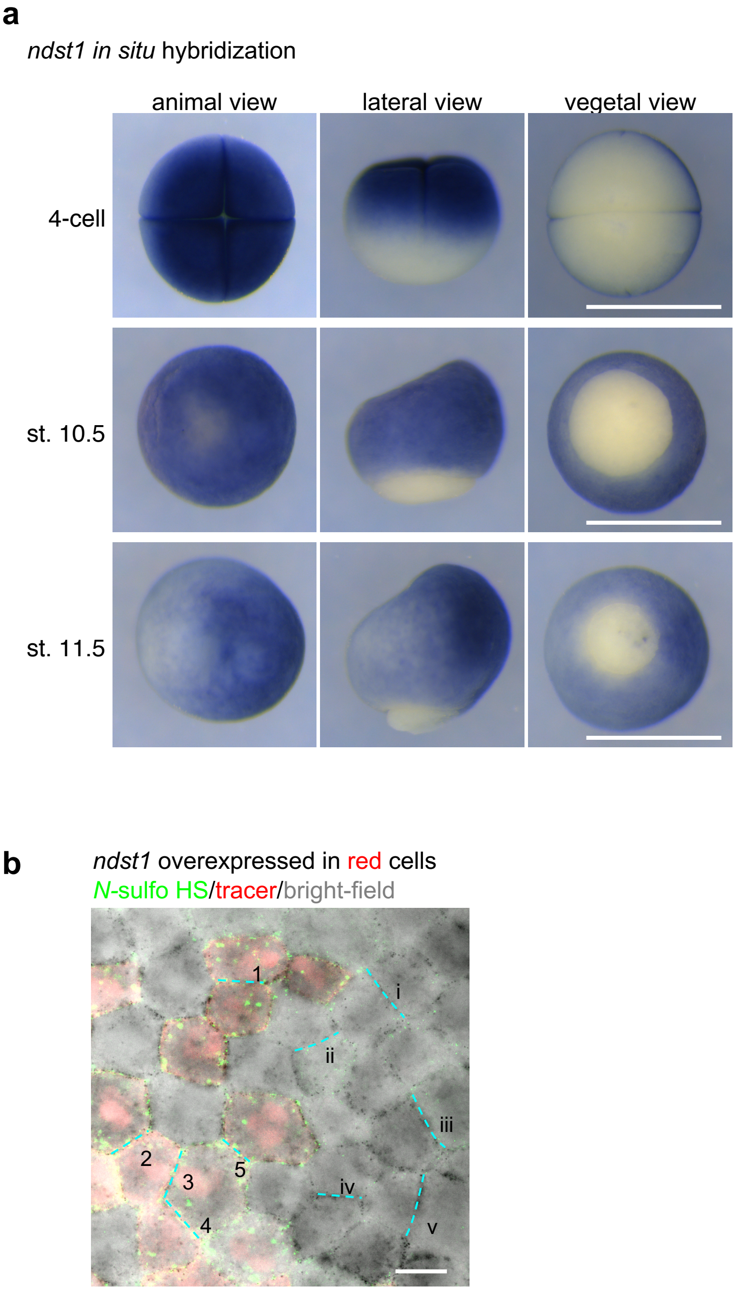

Supplementary Figure 4. Expression patterns of *Xenopus ndst1*.

a, Spatial distribution of *ndst1* mRNA in early *Xenopus* development. *ndst1* mRNA was visualized by whole-mount *in situ* hybridization using albino embryos of stage 3 (4-cell), stage 10.5 (early gastrula) or stage 11.5 (mid-gastrula). Views are shown as indicated with the dorsal to the right. During the gastrula stage, *ndst1* was expressed with a gradient from the dorsal to the ventral. Images are a representative of more than five embryos.

b, An example of quantification at cell boundaries. *N*-sulfo HS staining merged with tracer and bright field image in an Ndst1 overexpressing embryo is shown (see Fig. 3d). Same number of cell boundaries between two tracer-positive or -negative cells were randomly chosen. When merged with bright field image, cell boundaries are easily recognized but the fluorescent signal to be measured is rather hard to recognize, contributing to a random selection. Image J was used for quantification.

Scale bars, 1.0 mm (**a**) or 20 μm (**b**).

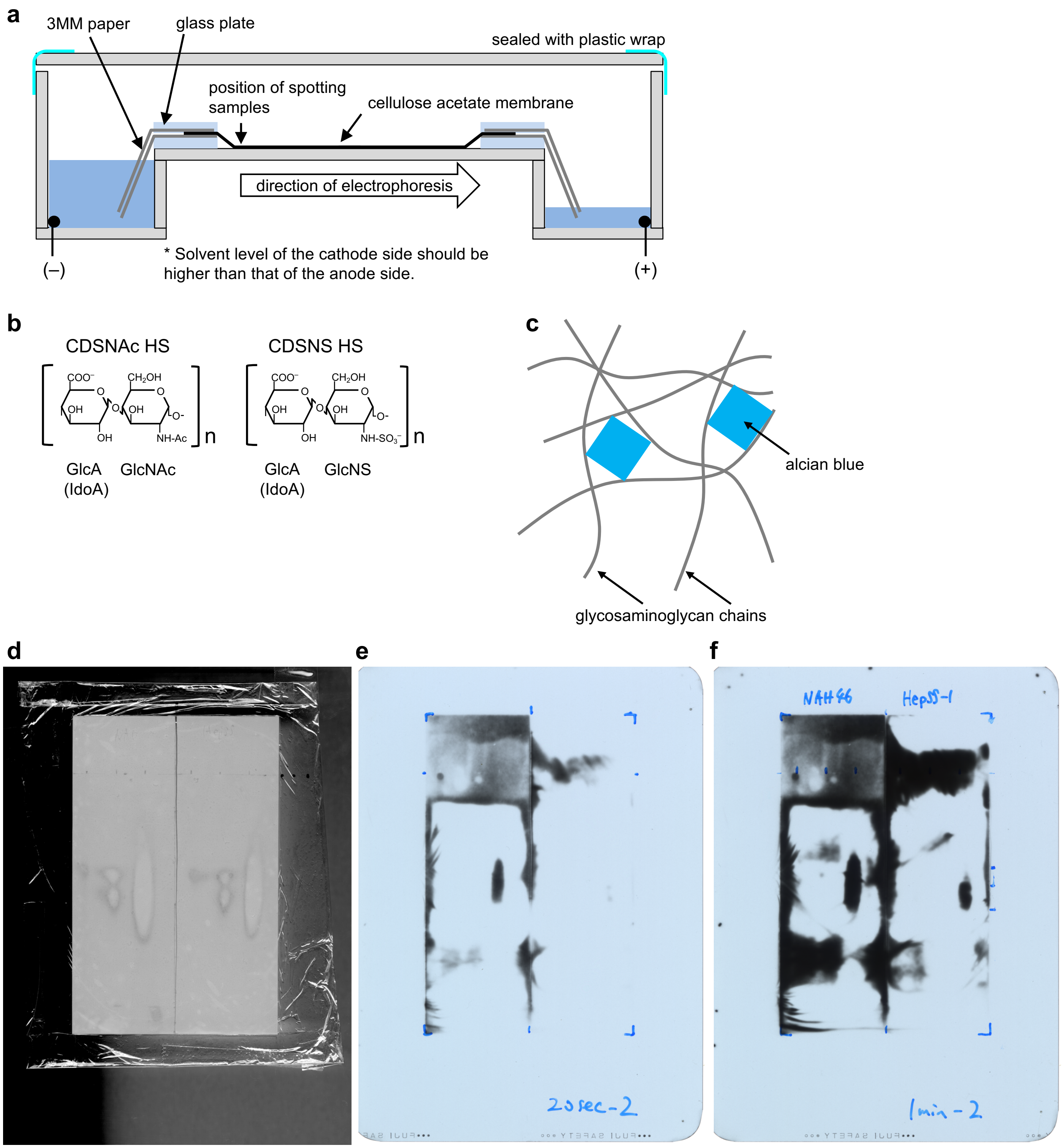

Supplementary Figure 5. Cellulose acetate membrane electrophoresis coupled with alcian-immunostaining.

a, Schema for cellulose acetate membrane electrophoresis (CAME).

b, Structures of CDSNAc HS and CDSNS HS. Note that these HS chains contain IdoA, which was epimerized from GlcA, in some portions, because those were chemically converted from natural HS from the bovine kidney.

c, Schema for fixation/staining of GAGs by alcian blue. GAGs stained with alcian blue become insoluble, which is probably due to bridging of multiple GAG chains.

d-f, Original scanned images of the cellulose acetate membrane stained with alcian blue (d) and the X-ray film for alcian-immunostaining with a short exposure (20 sec, e) or a long exposure (1 min, **f**). See also Fig. 3g.

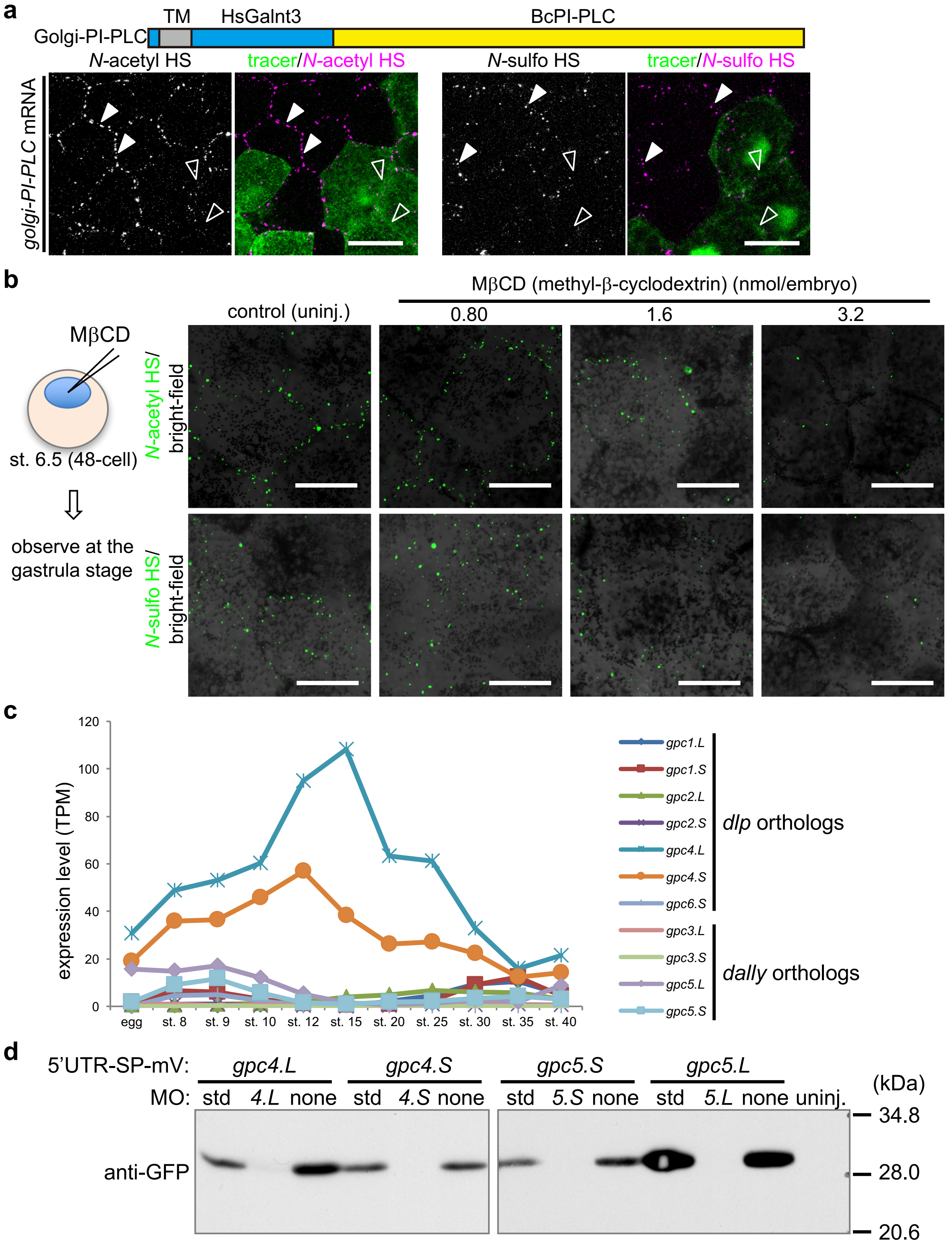

Supplementary Figure 6. Glypicans as candidates for core proteins of the HS clusters.

a, Reduction of the HS clusters by overexpression of Golgi-tethered PI-PLC (Golgi-PI-PLC). To examine whether PI-PLC can inhibit HS clusters formation in a cell-autonomous manner, a coding sequence of PI-PLC was synthesized, based on the protein sequence of *Bacillus cereus* PI-PLC and its Golgi-tethered construct was made with partial sequence of *Homo sapience galnt3* gene. mRNAs for Golgi-PI-PLC and Venus-NLS as a tracer were microinjected into a ventral blastomere at the 4-cell stage. Embryos were fixed at stage 11.5, and immunostained for *N*-acetyl-rich or *N*-sulfo-rich HS clusters as indicated. Scale bars, 20 μm.

b, Dose-dependent reduction of the HS clusters by methyl-β-cyclodextrin (MβCD) treatment. MβCD was introduced by blastocoel injection. Embryos were fixed and immunostained at stage 11.5. Scale bars, 20 μm.

c, Expression levels of *gpc* genes during embryogenesis of *Xenopus laevis*. The RNA-seq data were obtained from the *X. laevis* genome project[3,4]. Transcripts per million (TPM) were plotted for indicated genes and stages.

d, Inhibition of translation of *gpc* mRNAs by their MOs. Embryos were injected with MOs and mRNAs consisting of the 5' UTR, the signal peptide (SP) region of *gpc4.L*, *gpc4.S*, *gpc5.L* or *gpc5.S* and *mVenus*. The translated products were assayed by immunoblotting with anti-GFP antibody. Lysates equivalent to one embryo were applied per lane. MO for each *gpc* gene (*4.L*, *4.S*, *5.L*, or *5.S*) specifically blocked translation from its own mRNA while std MO, a negative control, did not.

Images are a representative of at least two independent experiments. Amounts of mRNAs (pg/embryo): *Golgi-PI-PLC*, 1.0; *venus-NLS*, 200; *5'UTR-SP-mV*, 1000. Amounts of MOs (ng/embryo): *gpc4.L*, 6.9; *gpc4.S*, 7.1; *gpc5.L*, 7.0; *gpc5.S*, 7.0; std MO, 6.9 (these are equimolar).

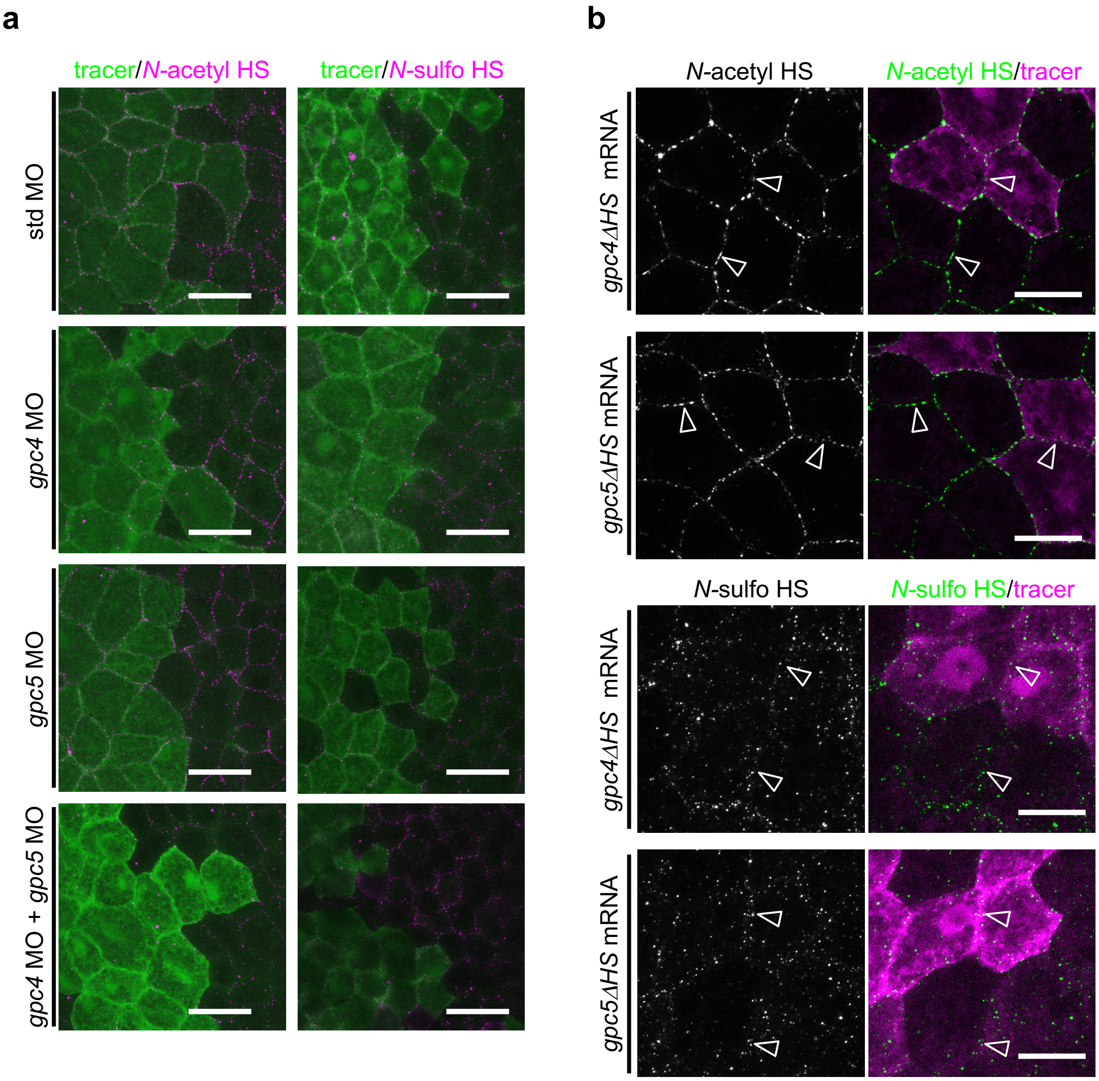

Supplementary Figure 7. ΔHS mutants of Gpc4 and Gpc5.

a, Separate images of tracer and HS staining for Fig. 4b.

b, No effects of ΔHS mutants on the HS clusters. mRNAs for the ΔHS mutant of Gpc4 or Gpc5, which lacks putative HS-attachment sites, and mRFP as a tracer were microinjected into 4-cell stage *Xenopus* embryo. Neither ΔHS mutants of Gpc4 or Gpc5 increased *N*-acetyl-rich nor *N*-sulfo-rich HS clusters (open arrowheads). Microinjection and observation were performed similarly to Fig. 4**c**.

Images are a representative of at least two independent experiments. Amounts of mRNAs (ng/embryo): *gpc4ΔHS* and *gpc5ΔHS*, 0.050; *mRFP*, 0.40. Scale bars, 40 µm (**a**); 20 µm (**b**).

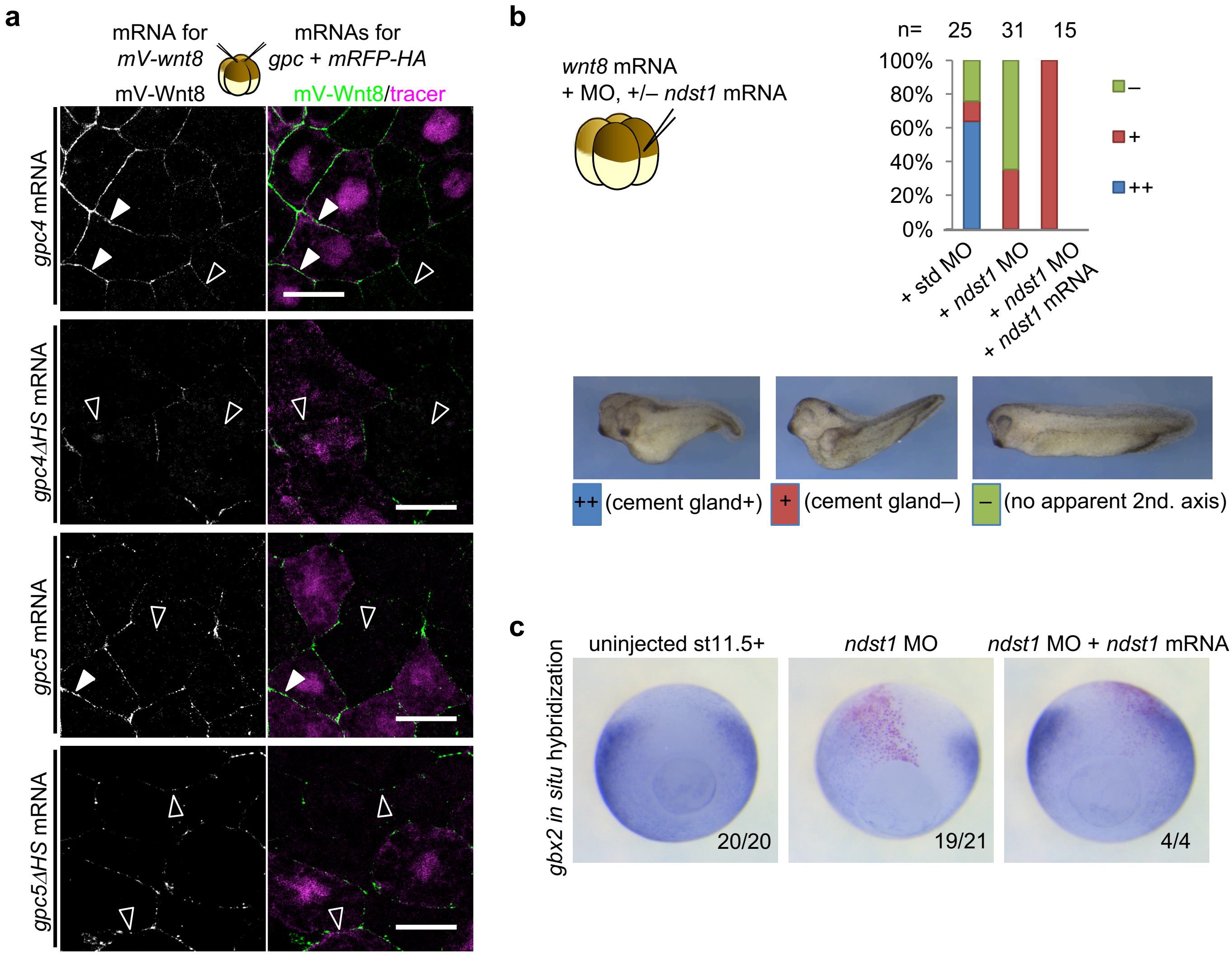

Supplementary Figure 8. *N*-sulfo-rich HS-dependent accumulation of Wnt8 on *gpc*-expressing cells and *ndst1* is required for canonical Wnt signalling.

a, HS-dependent accumulation of Wnt8. mRNAs for mV-Wnt8 and the wildtype or ΔHS mutant of Gpc4 or Gpc5 with mRFP as a tracer were separately microinjected into different blastomeres. Injected embryos were fixed and stained at stage 11.5. White arrowheads indicate the increase of mV-Wnt8 at the cell boundary, compared with open arrowheads in the same panel. Two open arrowheads in the same panel indicate no significant increase. Images are a representative of at least two independent experiments. Scale bars, 20 µm.

b, Inhibition of secondary axis formation by knockdown of *ndst1*. *ndst1* but not std MO inhibited secondary axis formation by ventral injection of *wnt8* mRNA at the 4-cell stage, which was rescued by coinjection with *ndst1* mRNA. Numbers of analysed embryos (n) were as indicated.

c, Reduction of *gbx2* expression by knockdown of *ndst1*. Embryos were coinjected with *ndst1* or std MO and nβ-gal mRNA into the dorsoanimal region at 4-cell stage, and subjected at the gastrula stage (st. 11.5-12) to whole-mount *in situ* hybridization for *gbx2*, a direct target of canonical Wnt signalling[5]. Injected side was traced by red-gal staining (red). *ndst1* MO inhibited expression of *gbx2*, which was rescued by injection of *ndst1* mRNA. Number of embryos with the shown phenotype/Number of analysed embryos is as indicated (bottom right of each panels).

Amounts of mRNAs (pg/embryo): *gpc4*, *gpc5*, *gpc4ΔHS* and *gpc5ΔHS*, 50; *mRFP*, 400, *mV-wnt8*, 1000; *wnt8*, 5; *ndst1*, 1.7 (**b**) or 5 (**c**); *nβ-gal*, 50. Amounts of MOs, 14 ng/embryo.

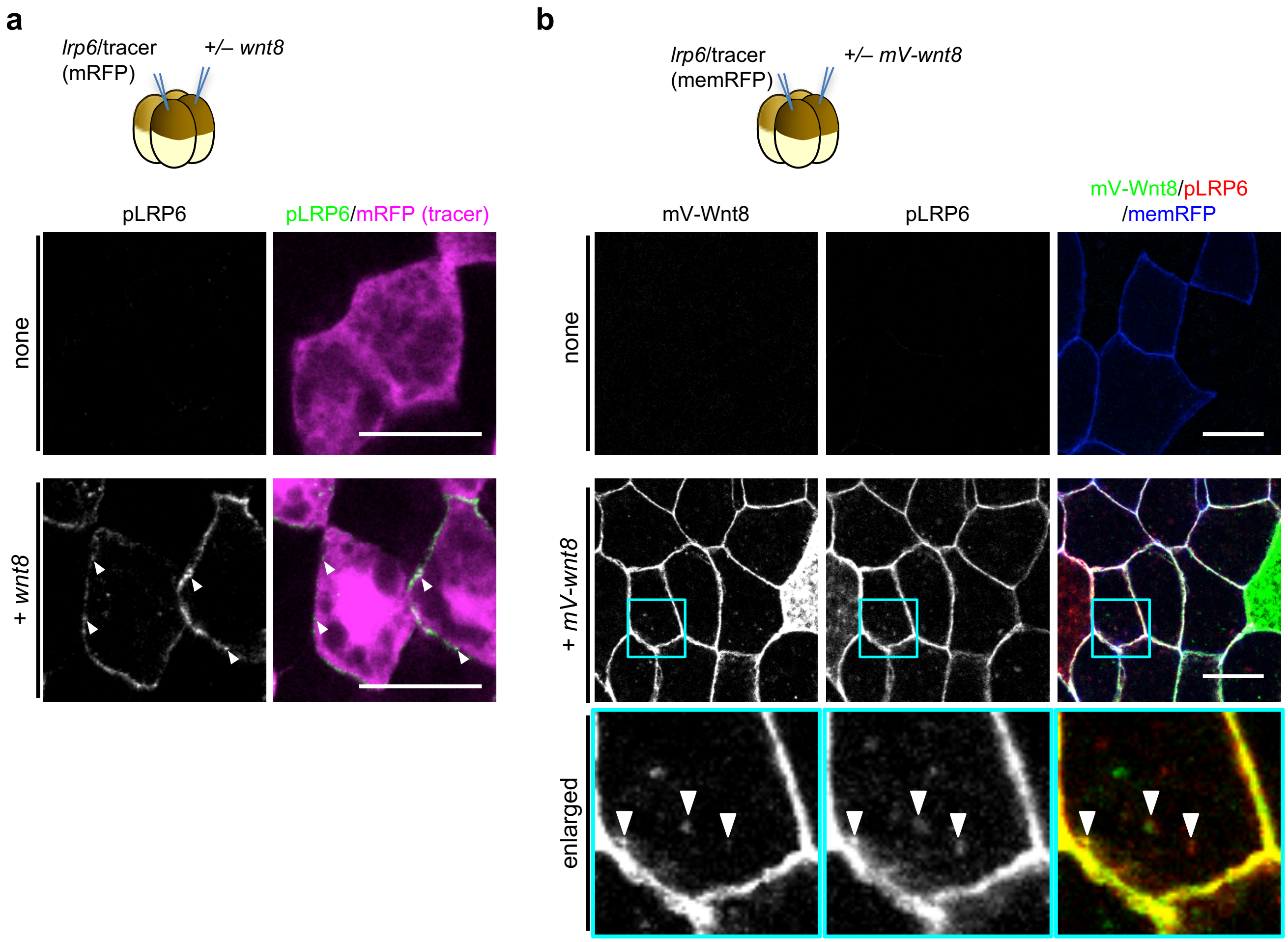

Supplementary Figure 9. Wnt8-dependent phosphorylation of LRP6.

a, Wnt8-dependent phosphorylation of LRP6. Staining of phosphorylated LRP6 (pLRP6) was observed in a Wnt8 dependent manner. Note that the distribution of pLRP6 shows puncta (arrowheads) similar to that of Wnt8.

b, Internalization of pLRP6 with mV-Wnt8. Puncta of pLRP6 inside cells were colocalised with those of mV-Wnt8 (arrowheads). pLRP6 staining was mV-Wnt8-dependent again, however, the distribution of pLRP6 on the cell boundaries are rather continuous, due to the higher dose of mV-Wnt8 than that of Wnt8 in **a**.

Images are a representative of at least two independent experiments. Amounts of mRNAs (pg/embryo): *lrp6-vsvg,* 125 (a) or 42 (b)*;wnt8*, 125; *mRFP*, 500, *mV-wnt8*, 1000; *memRFP*, 100. Scale bars, 20 μm.

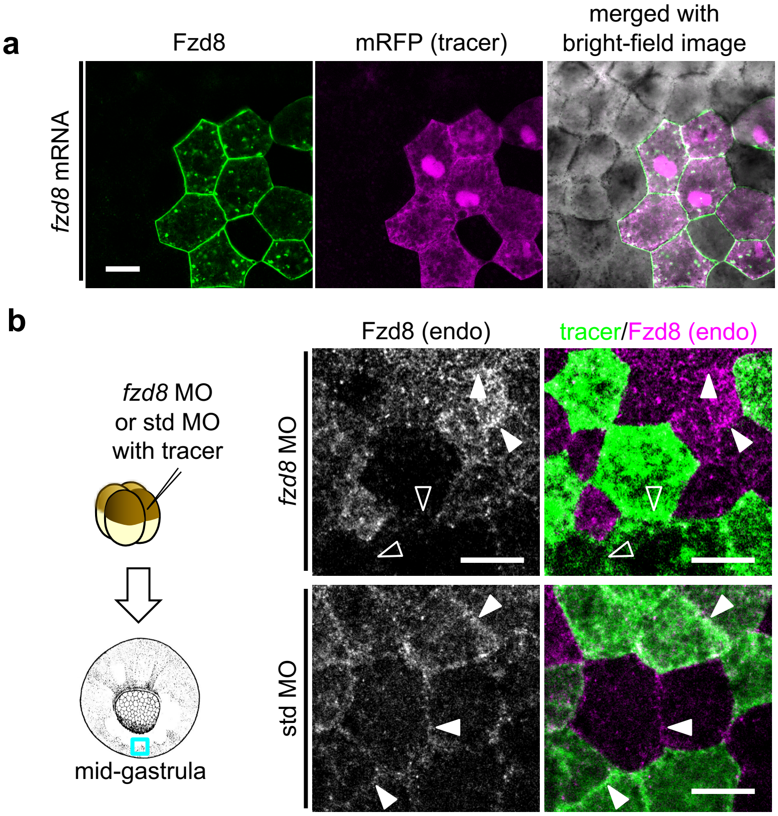

Supplementary Figure 10. Validation of anti-Fzd8 antibody.

a, Specificity of anti-Fzd8 antibody. *Xenopus fzd8* mRNA was injected into the animal pole region of a ventral blastomere together with *mRFP* mRNA as a tracer. Injected embryos were fixed at st. 11.5, and stained with anti-Fzd8 antibody. Confocal images showed that plasma membrane of the tracer-positive cells was strongly stained, suggesting this antibody specifically recognizes *Xenopus* Fzd8 protein

b, *fzd8* MO was injected into ventral equatorial region, where corresponds to the future VMZ, and the superficial layer of the VMZ was observed at mid-gastrula stage (st. 11.5). Observed region is roughly indicated by the cyan box. *fzd8* MO reduced the staining with the anti-Fzd8 antibody at the cell boundary (open arrowheads) and inside cells, whereas a negative control, std MO did not (white arrowheads, for cell boundary), suggesting that the anti-Fzd8 antibody recognizes endogenous Fzd8.

Images are a representative of two independent experiments. Amounts of mRNAs (pg/embryo): *fzd8*, 1000; *mRFP*, 500. Amounts of MOs, 14 ng/embryo. Scale bars, 20 µm.

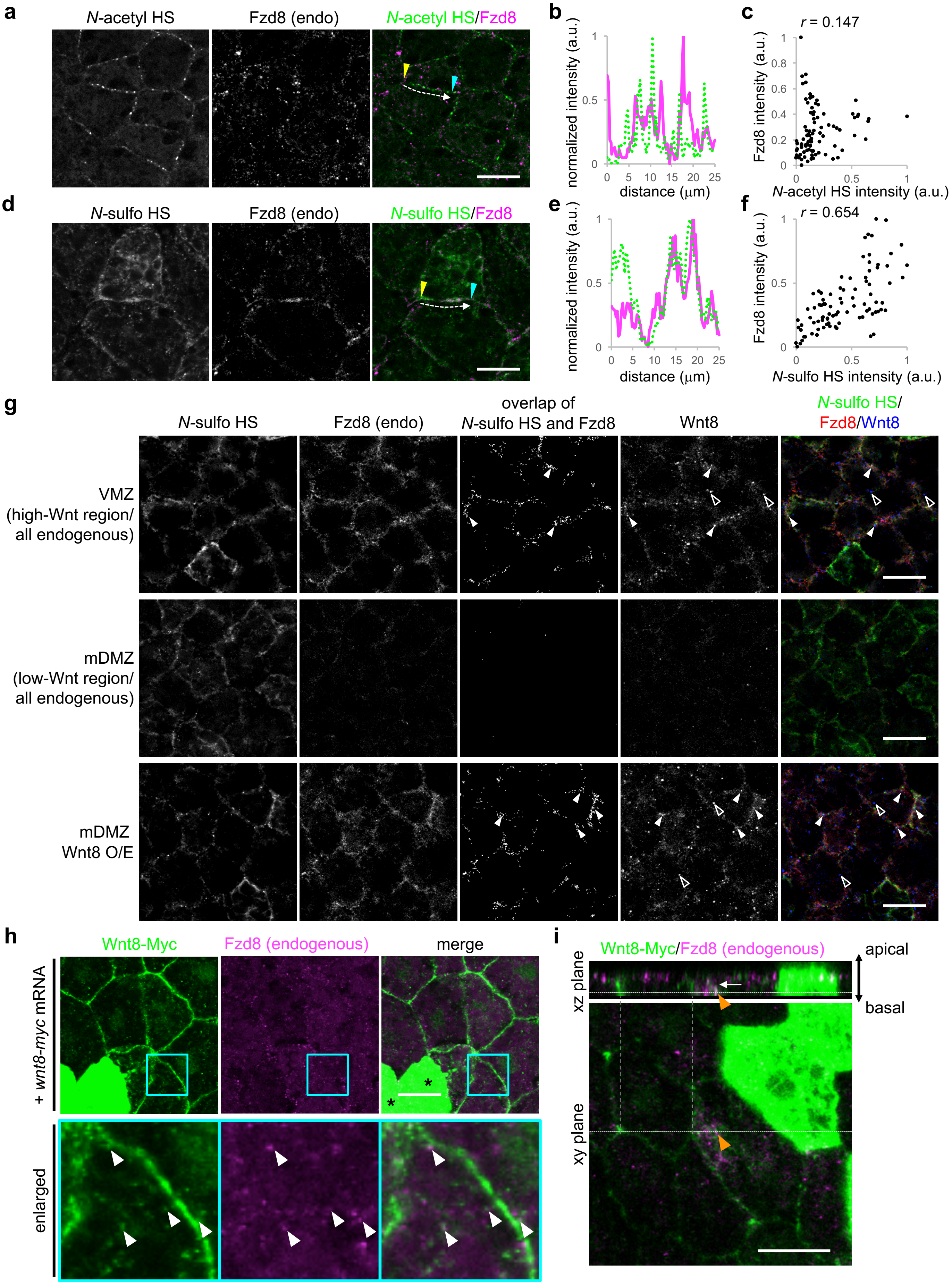

Supplementary Figure 11. Endogenous Fzd8 as a component of the Frizzled/Wnt/LRP6 signalosomes.

a-f, The colocalisation of endogenous Fzd8 and *N*-sulfo-rich HS clusters. st. 11.5 embryos were co-immunostained for Fzd8 and *N*-acetyl (**a**) or *N*-sulfo HS (**d**). Signal intensities along white arrows were plotted (**b**,**e**), starting and ending points as indicated by yellow and cyan arrowheads, respectively (**a**,**d**). Distributions of Fzd8 and *N*-acetyl HS were not correlated well (correlation coefficient $r = 0.147$), whereas distributions of Wnt8 and *N*-sulfo HS were highly correlated ($r = 0.654$) (**c**,**f**). Thus, Fzd8 and *N*-sulfo-rich HS clusters are highly colocalised on the membrane.

g, Co-immunostaining of *N*-sulfo HS, Fzd8 and Wnt8. Immunostaining was carried out for detecting all endogenous molecules except for overexpressed Wnt8. Overlap of *N*-sulfo HS and Fzd8 (3rd column from the left, see method for calculation of "overlap") were positively correlated with the amounts of endogenous Wnt8 protein (compare VMZ and mDMZ) and exogenous Wnt8. Also, colocalisation of *N*-sulfo HS, Fzd8, and Wnt8 were observed (arrowheads). Some puncta of Wnt8 did not colocalised with *N*-sulfo HS and Fzd8 (open arrowheads).

h, Internalization of Fzd8 with Wnt8-Myc. Source cells of Wnt8-Myc are marked with asterisks. Note that puncta of Wnt8-Myc inside cells near Wnt8-Myc expressing cell (asterisk) are considered to be internalized. Internalized puncta of Wnt8-Myc were colocalised with Fzd8 (arrowheads).

i, Optical section for XZ plane to confirm the depth of the observation. Overlapping puncta of Wnt8-Myc and Fzd8 (arrowhead) is inside cell, not continuous from the surface (arrow).

Images are a representative of at least two independent experiments. Amounts of injected mRNAs (pg/embryo): *wnt8*, 250; *wnt8-myc*, 250. Scale bars, 20 μm. a.u., arbitrary units.

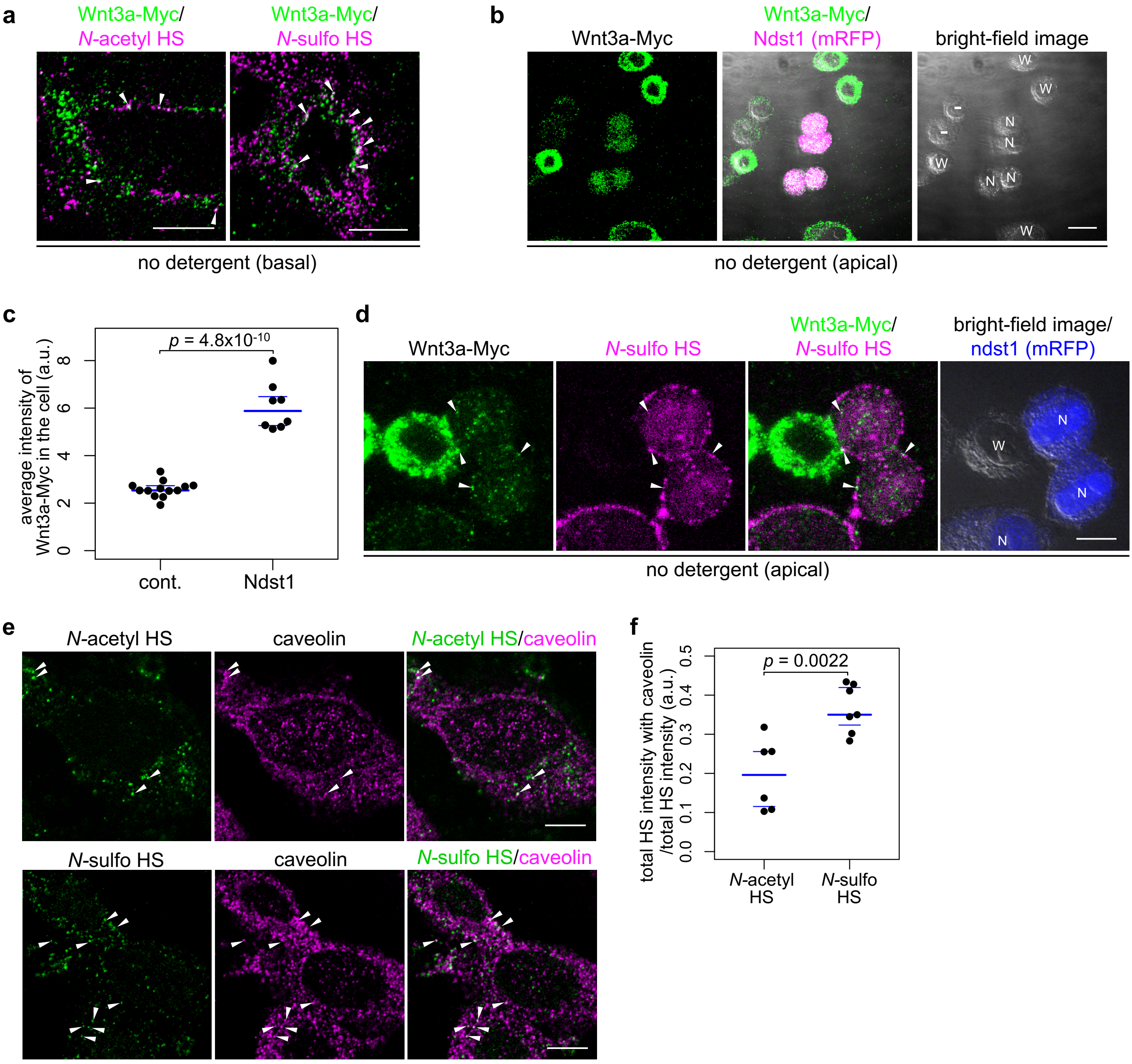

Supplementary Figure 12. Wnt3a and signalosome components were localised with *N*-sulfo-rich HS in HeLa cells.
a-d, Colocalisation of Wnt3a with *N*-sulfo-rich HS. **a**, Distribution of Wnt3a-Myc and *N*-acetyl/*N*-sulfo-rich clusters in Wnt3a-Myc expressing cells. To detect the secreted Wnt3a-Myc, permeabilization was not performed. Wnt3a-Myc (green) was well colocalised with *N*-sulfo HS (magenta) (arrowheads), observed at the basal side of the cells. **b**, Wnt3a-Myc was localised on Ndst1-expressing cells. Wnt3a-Myc (green) and Ndst1 with mRFP (magenta) were transfected to the cells in the different dishes and then these cells were co-cultured for 17 hrs. In the bright-field image, "W" indicates Wnt3a-Myc expressing cells; "N", Ndst1-expressing cells; "–", untransfected cells. To detect the secreted Wnt3a-Myc, permeabilization was not performed. Wnt3a-Myc was well localised on Ndst1-expressing cells, compared with non-expressing cells, observed at the apical side of the cells. **c**, Quantification of average signal intensities of Wnt3a-Myc on Ndst1-expressing or untransfected cells (cont.) in **b**. Ndst1-expressing cells showed a significantly higher localisation of Wnt3a-Myc than untransfected control cells (*t*-test). The horizontal lines indicate first quartile, median, third quartile, respectively. **d**, Wnt3a-Myc was localised on *N*-sulfo-rich HS clusters in Ndst1-expressing cells at the apical side of the cells. To detect the secreted Wnt3a-Myc, permeabilization was not performed. Arrowheads indicate colocalisation of Wnt3a-Myc (green) and *N*-sulfo HS (magenta).
e,**f**, Immunostaining of caveolin with *N*-acetyl HS or *N*-sulfo HS. **e**, *N*-sulfo HS (green) was well colocalised with caveolin (magenta) (arrowheads). **f**, Quantification of colocalisation of *N*-acetyl HS with caveolin and *N*-sulfo HS with caveolin, represented by the Manders' coefficients[6,7], which were calculated automatically using Fiji (ImageJ) software. Manders' coefficients of *N*-sulfo HS and caveolin were significantly higher than those of *N*-acetyl HS and caveolin (*t*-test). Graphs were written with R using the R package, beeswarm, (**c**, **f**). Images are a representative of at least two independent experiments. Scale bars, 10 µm. a.u., arbitrary units.

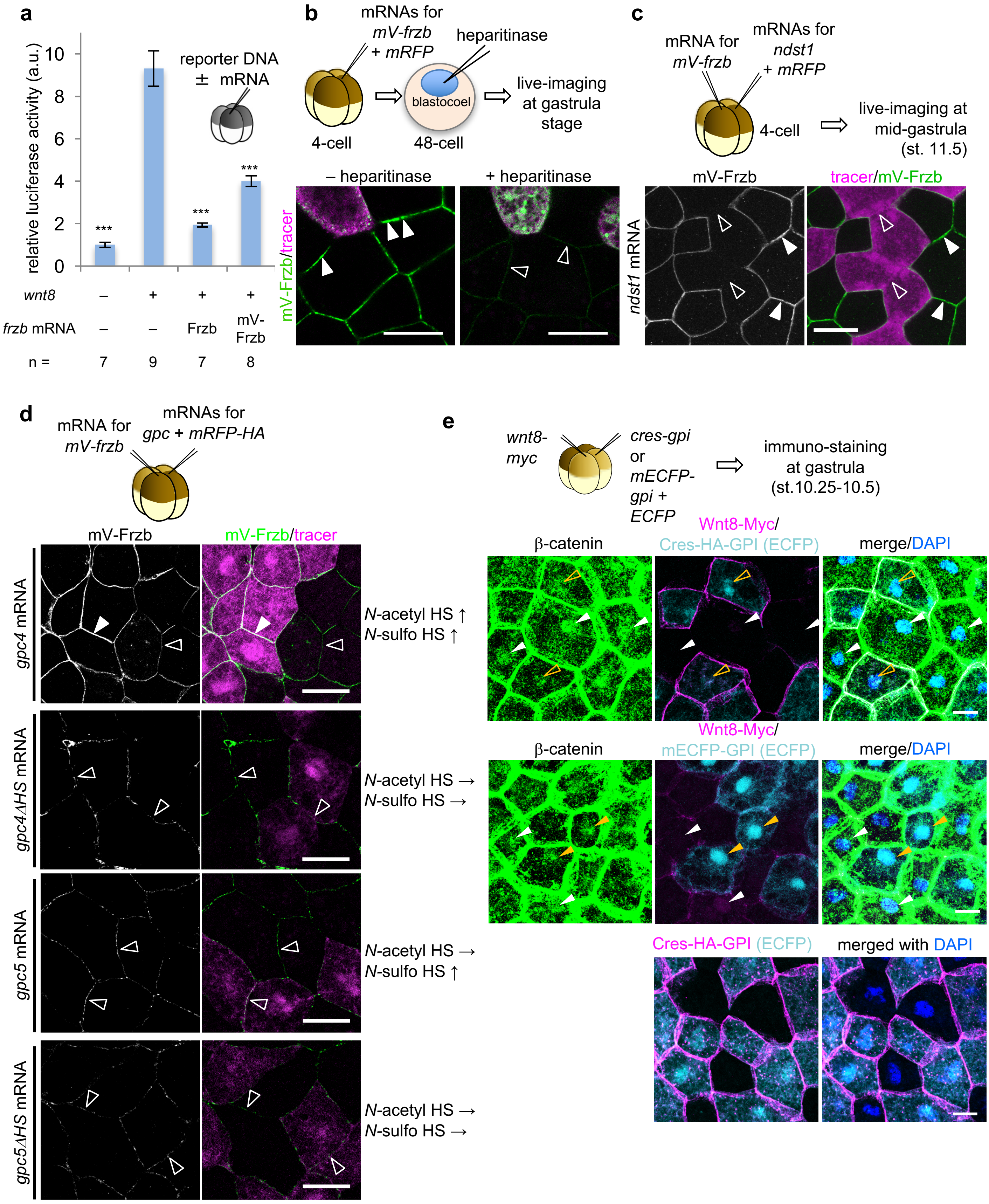

Supplementary Figure 13. ***N*-acetyl-rich HS clusters retain Frzb on the cell surface.**

a, Biological activity of mV-Frzb as evaluated by luciferase reporter assays for inhibition of canonical Wnt signalling. Statistical significance, $***p < 0.001$, pairwise Wilcoxon rank sum test (two-sided); error bars, s.e.m.. The numbers of pools (each pool contains 3 embryos) are indicated at the bottom. a.u., arbitrary unit.
b, HS chain-dependent distribution of Frzb in the extracellular space. Experimental procedures were as illustrated on the top. Left, mV-Frzb showed intercellular distribution with somewhat continuous puncta (white arrowheads). Right, blastocoel-injected heparitinase reduced the intercellular distribution of mV-Frzb (open arrowheads).
c, Inhibition of Frzb localisation at the cell boundary with Ndst1 overexpression. Experimental procedures were as illustrated on the top. mV-Frzb in the intercellular space was clearly reduced between *ndst1*-overexpressing cells (open arrowheads against white arrowheads).
d, *N*-acetyl HS-dependent accumulation of Frzb on *gpc4*-overexpressing cells. mRNAs for mV-Frzb and Gpc4/5 with mRFP as a tracer were separately microinjected into different blastomeres as illustrated. White arrowheads indicate increase of mV-Wnt8 at the cell boundary, compared with the open arrowhead in the same panel. Two open arrowheads in the same panel indicate no significant increase. Expected increase (↑), decrease (↓) and no change (→) of *N*-sulfo/*N*-acetyl-rich HS clusters are as indicated on the right.
e, Effect of the expression of GPI-anchored Crescent on Wnt signalling. Injection of GPI-anchored Crescent (top panels; Cres-HA-GPI) accumulated Wnt8-myc but not enhanced nuclear accumulation of β-catenin (yellow arrowheads), compared with uninjected cells (white arrowheads). Open and closed arrowheads indicate absence/reduction and presence, respectively, of nuclear β-catenin staining. GPI-anchored mECFP was used as a negative control. Membrane localisation of Cres-HA-GPI was confirmed by HA staining (lower panels).
Images are a representative of at least two independent experiments. Amount of the TOP-FLASH reporter DNA, 200 pg/embryo. Amounts of injected mRNAs (pg/embryo): *wnt8*, 25; *frzb*, 10; *mV-frzb*, 15.7 (**a**), 1000 (**b, c**) or 500 (**d**); *mRFP*, 500, *ndst1*, 500; *gpc4, gpc5*, *gpc4ΔHS* and *gpc5ΔHS*, 50; *mRFP*, 400. Amounts of *frzb* and *mV-frzb* were equimolar in **a**. Scale bars, 20 μm.

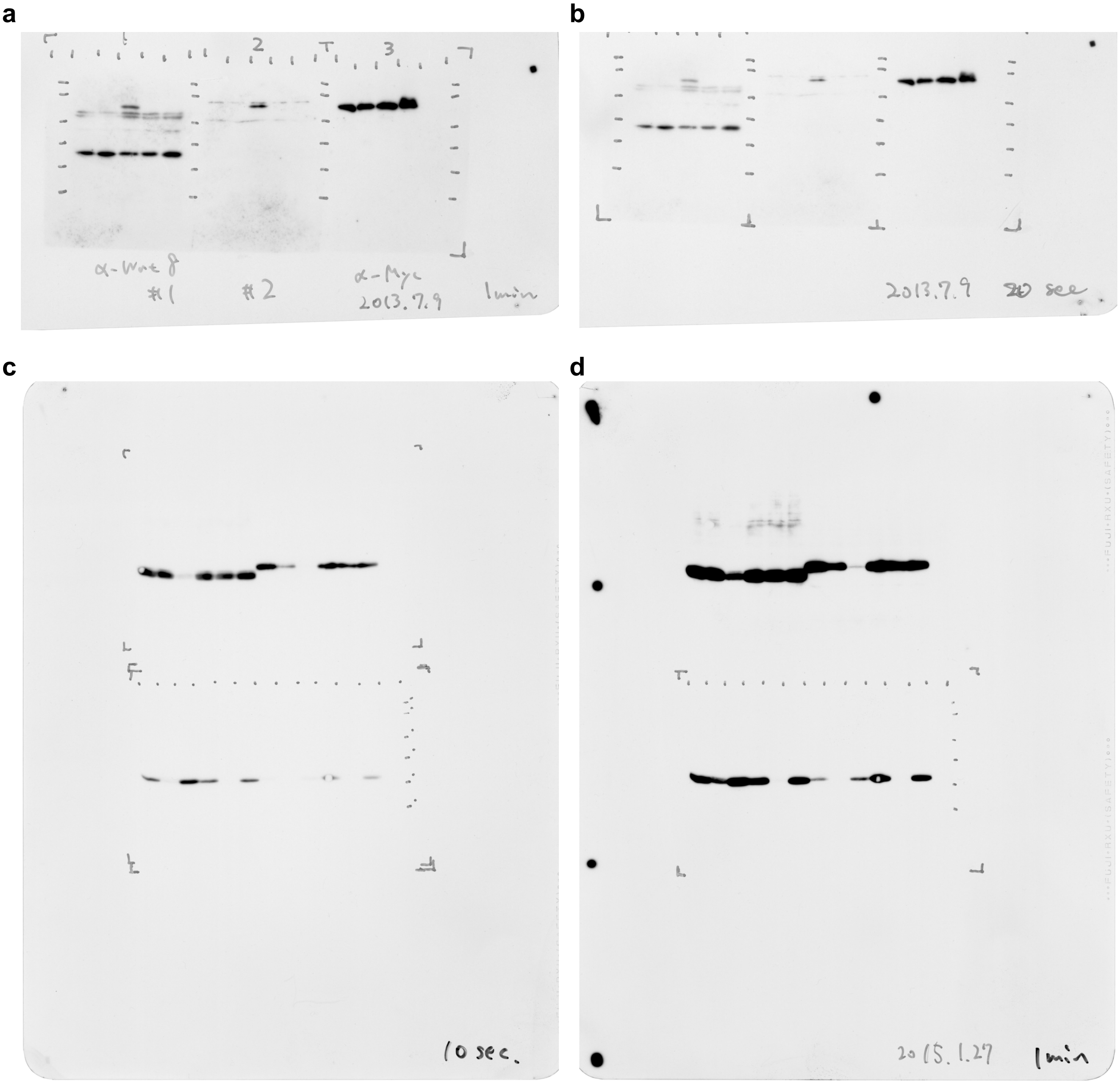

Supplementary Figure 14. Uncropped images of western blotting.
Uncropped scanned images of western blotting for Supplementary Fig. 1b, anti-Wnt8 (a), anti-Myc (b) and Supplementary Fig. 6d, left (c), right (d).

Supplementary references

1. Kure, S. & Yoshie, O. A syngeneic monoclonal antibody to murine Meth-A sarcoma (HepSS-1) recognizes heparan sulfate glycosaminoglycan (HS-GAG): cell density and transformation dependent alteration in cell surface HS-GAG defined by HepSS-1. *J. Immunol.* **137**, 3900-8 (1986).
2. Czajkowsky, D. M. & Shao, Z. The human IgM pentamer is a mushroom-shaped molecule with a flexural bias. *Proc. Natl Acad. Sci. USA* **106**, 14960-5 (2009).
3. Session, A. M. et al. Genome evolution in the allotetraploid frog Xenopus laevis. *Nature* **538**, 336-343 (2016).
4. Michiue, T. et al. High variability of expression profiles of homeologous genes for Wnt, Hh, Notch, and Hippo signaling pathways in Xenopus laevis. *Dev. Biol.* **426**, 270-290 (2016).
5. Li, B., Kuriyama, S., Moreno, M. & Mayor, R. The posteriorizing gene Gbx2 is a direct target of Wnt signalling and the earliest factor in neural crest induction. *Development* **136**, 3267-78 (2009).
6. Manders, E. M. M., Verbeek, F. J. & Aten, J. A. Measurement of co-localisation of objects in dual-colour confocal images. *J. Microsc.* **169**, 375-382 (1993).
7. Chmiest, D. et al. Spatiotemporal control of interferon-induced JAK/STAT signalling and gene transcription by the retromer complex. *Nat. Commun.* **7**, 13476 (2016).
