# Supplementary material for: Roles of two types of heparan sulfate clusters in Wnt distribution and signaling in Xenopus
Source: Nat Commun. 2017 Dec 7;8:1973. doi: 10.1038/s41467-017-02076-0 (PMC5719454; doi:10.1038/s41467-017-02076-0)
Supplement: Supplementary file 3 — Description of Additional Supplementary Files [file 41467_2017_2076_MOESM3_ESM.docx]

**Description of Additional Supplementary Files**

File Name: Supplementary Movie 1

Description: Internalization of mV-Wnt8 in the Xenopus embryo. Internalization of mVenus (mV)-Wnt8 puncta at the cell boundary was observed in the animal cap region of the *Xenopus* gastrula (st. 11.5). Gradually, a budding occurred at the most apparent puncta and then internalized into the upper side cell. The observed region was vicinity of mV-Wnt8 expressing cells, whose inside looks white. The play speed is x10. See the main text and Fig. 7a for the detail.
